# Glycogen Synthase Kinase-3 Regulates Sperm Motility and Acrosome Reaction via Affecting Energy Metabolism in Goats

**DOI:** 10.3389/fphys.2019.00968

**Published:** 2019-07-30

**Authors:** Zhendong Zhu, Rongnan Li, Liqiang Wang, Yi Zheng, S. A. Masudul Hoque, Yinghua Lv, Wenxian Zeng

**Affiliations:** ^1^Key Laboratory of Animal Genetics, Breeding and Reproduction of Shaanxi Province, College of Animal Science and Technology, Northwest A&F University, Yangling, China; ^2^Department of Animal Breeding and Genetics, Bangabandhu Sheikh Mujibur Rahman Agricultural University, Gazipur, Bangladesh; ^3^College of Chemistry & Pharmacy, Northwest A&F University, Yangling, China

**Keywords:** goat, GSK3α/β, metabolism, motility, sperm

## Abstract

Hyperactivation and acrosome reaction of sperm are pre-requisite steps for fertilization. However, the hyperactivation and acrosome reaction are critically controlled through the phosphorylation of specific proteins. Glycogen synthase kinase-3 (GSK3), a serine/threonine kinase with two different isoforms (α and β), is involved in biochemical signaling pathways. This study was aimed to investigate whether the GSK3α/β is present in goat sperm and its regulatory role in sperm motility and acrosome reaction. GSK3α/β was detected with immunofluorescence and Western blotting. Sperm motility, membrane integrity, acrosome reaction, mitochondrial membrane potential, phospho-Ser21-GSK3α and phospho-Ser9-GSK3β were analyzed. The ATP production and activities of lactate dehydrogenase (LDH), malate dehydrogenase (MDH), and succinate dehydrogenase (SDH) were measured. It was observed that the GSK3α/β was expressed in goat sperm, especially in the peri-acrosomal, mid-piece and principal piece of the tail. The abundance of GSK3α/β in sperm was increased during transit along the epididymis. Addition of either 5-aminoimidazole-4-carboxamide ribonucleotide (AICAR) or CHIR99021 significantly increased the sperm motility patterns and GSK3α/β phosphorylation. Interestingly, the adenosine triphosphate (ATP) production, activities of LDH, MDH and SDH were observed to be increased in the CHIR99021 treatment. The results suggested that GSK3α/β regulates sperm motility and acrosome reaction via phospho-ser21-GSK3α and phospho-ser9-GSK3β that involved in the regulation of sperm energy metabolism.

## Introduction

Sperm are highly differentiated and specialized cells that transmit the genetic information to the next generation. Mammalian sperm are non-motile and unable to fertilize the oocytes just after released from the epithelia of seminiferous tubules in testes. During the migration from caput to caudal epididymis, sperm become mature and achieve motile ability (Dacheux and Dacheux, 2014). Sperm must undergo a sequential series of complex process in the uterus and oviduct before fertilizing the oocytes, including capacitation and acrosome reaction (Suarez and Pacey, 2006; Gervasi and Visconti, 2016) that can be mimicked *in vitro* using bicarbonate, calcium, heparin and bovine serum albumin (BSA) (Stival et al., 2016). ATP production through the glycolysis and oxidative phosphorylation (OXPHOS) is essential for sperm motility and acrosome reaction (Stival et al., 2016). Therefore, the activation of the kinases that regulate cellular ATP production was suggested to involve in governing the sperm functions (Nguyen, 2017; Zhu et al., 2018).

Glycogen synthase kinase 3 (GSK3), a serine/threonine protein kinase, is involved in the biochemical pathways mediating cell metabolism (Kaidanovich-Beilin and Woodgett, 2011). Two GSK3 isoforms, α (51KD) and β (47KD) that are encoded by two independent genes *gsk3α* and *gsk3β*, have been identified in mammalian cells (Woodgett, 1990). The main difference between GSK3α and GSK3β is the presence of a glycine-rich area in the N-terminus of GSK3α (Woodgett, 1990; Frame and Cohen, 2001). GSK3 is phosphorylated on Tyr279 (GSK3α), Tyr216 (GSK3β) or Ser21 (GSK3α), and Ser9 (GSK3β) (Medina et al., 2011). It has been reported that the GSK3 serine phosphorylation leads to inactivate the GSK3 protein and consequently, increases the downstream target-proteins activity (Beurel et al., 2015). In addition, previous studies reported that both GSK3α and β isoforms were present in the sperm of boars (Aparicio et al., 2007), bulls (Somanath et al., 2004), and mice (Bhattacharjee et al., 2015; Reid et al., 2015). Somanath et al. (2004) observed that serine phosphorylation of the GSK3 in motile caudal sperm was sixfold higher than those in non-motile caput sperm in bulls. Interestingly, when the calyculin A was used to incubate with bovine sperm to increase the motility *in vitro*, the level of GSK3 serine phosphorylation was significantly increased compared to the control (Somanath et al., 2004). Moreover, Aparicio et al. (2007) observed that the activation of serine phosphorylation in GSK3α with alsterpaullone significantly improved the velocity parameters of boar sperm. On the other hand, Bhattacharjee et al. (2015) reported that the targeted mutation of *gsk3α* significantly reduced mouse sperm motility *in vivo*. Those phenomena indicated that the GSK3 serine phosphorylation was correlated with sperm motility (Aparicio et al., 2007). However, the underlying mechanism of GSK3 involved in the regulation of sperm motility is largely unknown.

In addition, the GSK3α/β protein not only played a crucial role in regulating mouse sperm maturation but also in acrosome reaction (Reid et al., 2015). However, the inhibition of GSK3 activity by alsterpaullone did not affect the number of acrosome-reacted boar sperm (Aparicio et al., 2007). Moreover, the information about the function of GSK3α/β in goat sperm is limited. Therefore, the aims of the present study were (i) to determine whether the GSK3α/β protein is present in goat sperm; (ii) to study whether and how the GSK3α/β regulates goat sperm motility and acrosome reaction.

## Materials and Methods

### Reagents and Medium

All chemicals were purchased from Sigma-Aldrich (St. Louis, MO, United States), unless specified. Tris-citric acid-glucose (TCG) extenders were prepared according to Moce et al. (2014), which composed of 250 mM Tris [hydroxymethyl] aminomethane, 83 mM of citric acid anhydrous and 69 mM of D-glucose; 300 mOsm, and pH = 7.0. Capacitation medium (CM) was prepared according to our previous study (Zhu et al., 2018) using 94.6 mM NaCl, 4.78 mM KCl, 5.56 mM D-glucose, 1.19 mM K_2_HPO_4_, 0.5 mM sodium pyruvate, 21.58 mM sodium lactate, 1.29 mM MgSO_4_, 5 mM CaCl_2_, 25 mM NaHCO_3_, 7 mg/mL BSA and 10 μg/mL heparin. Non-capacitation medium (NCM) was prepared according to Salmon et al. (2016), which consisted of 113.1 mM NaCl, 4.78 mM KCl, 5.56 mM D-glucose, 1.19 mM K_2_HPO_4_, 0.5 mM sodium pyruvate, 21.58 mM sodium lactate and 1.29 mM MgSO_4_, pH = 7.4 and 290 mOsm. Caudal epididymis sperm was collected and incubated *in vitro* in CM and NCM separately for 3 h at 38.5°C.

### Sperm Preparation

All animal and experimental procedures were approved by the Northwest A&F University Institutional Animal Care and Use Committee. Testes from mature goats with intact tunica were obtained from a local slaughterhouse. Briefly, the caput, corpus and cauda region of the epididymis were dissected and placed in a TCG extender. Several incisions were then made in each tissue, and sperm were gently teased out into the TCG extender with mild agitation. The resultant cell suspension was then layered over a 27% Percoll gradient and subjected to centrifugation at 400 × *g* for 15 min. A population of approximately 95% pure caput, corpus and cauda sperm was obtained from the pellet. Those sperm cells were then used for the following experiments.

### Epididymosomes Isolation

According to Reilly et al. (2016), the luminal fluid was aspirated from the cauda by placing the tissue in a 5 mL TCG medium and multiple incisions were done with a razor blade. The tissue was then subjected to mild agitation. Subsequently, the medium was filtered with 70 μm membranes. The suspension was used to isolate the epididymosomes with a Total Exosome Isolation Reagent (4484453; Thermo Fisher Scientific K.K., Waltham, MA, United States) following the manufacturer’s instructions. The epididymosomes were preserved at −80°C for Western blotting.

### Sperm Motility

Sperm motility was assessed with a computer-assisted sperm analysis system (CASA) (Integrated Semen Analysis System; Hview, Fuzhou, China). A total of 4 μL of semen samples were placed on a prewarmed (37°C) slides (CELL-VU^®^ DRM-600, Hamilton Throne, Beverly, MA, United States) and enclosed using a coverslip before immediately transferring to the CASA. The standard parameter settings were set at 25 frames/s, VCL > 10 μm/s to classify as sperm motile (Tamayo-Canul et al., 2011). A minimum of 300 sperm were observed from five randomly selected fields. Recorded parameters were: total motile sperm (%), progressively motile sperm (%), straight-line velocity (VSL, μm/s), curvilinear velocity (VCL, μm/s), average-path velocity (VAP, μm/s), linearity (LIN, %), beat-cross frequency (BCF, Hz) and wobble (WOB, %).

### Membrane Integrity

LIVE/DEAD Sperm Viability Kit (L7011; Thermo Fisher Scientific K.K.) was used to evaluate sperm membrane integrity (Zhu et al., 2017c). Briefly, sperm suspensions were stained with 100 nM SYBR-14 and 12 μM propidium iodide (PI). The staining was monitored and photographed with an epifluorescence microscope (80i; Nikon, Tokyo, Japan) with a set of filters (400×). The sperm were classified into three groups (Supplementary Figure 1A): membrane intact (blue arrow), membrane slightly damaged (white arrow) and membrane damaged (yellow arrow). All samples were identified and evaluated by one observer, and three replicates were assessed from each semen sample (*n* = 3).

### Acrosome Reaction Assessment With FITC-PNA/PI

The acrosome reaction was detected by 100 μg/mL fluorescein isothiocyanate-conjugated peanut agglutinin (FITC-PNA; Sigma, St. Louis, MO, United States) and 12 μM propidium iodide (PI) staining. According to the previous study (Zhu et al., 2017b), sperm samples were stained with FITC-PNA/PI, monitored and photographed with an epifluorescence microscope (80i; Nikon). As shown in Supplementary Figure 1B, the fluorescence images of sperm stained with FITC-PNA/PI could be classified into 2 groups: acrosome reaction (white arrow) and acrosome non-reaction (blue arrow). All samples were identified and evaluated by one observer, and three replicates were assessed from each semen sample (*n* = 3).

### Mitochondrial Membrane Potentials (Δψm)

The sperm mitochondrial membrane potential (Δψm) was analyzed using JC-1 (5,5′,6,6′-tetrachloro-1,1′,3,3′-tetraethylbenzimidazolo-carbocyanine iodide) Mitochondrial Membrane Potential Detection Kit (Beyotime Institute of Biotechnology, Nanjing, China) according to the previous studies (Chen et al., 2009; Zhu et al., 2017a). There are two types of JC-1 in stained mitochondrial plasma, one is the monomer, which emits green fluorescence in a low Δψm, and the other is the aggregate, which emits red fluorescence in a high Δψm. Briefly, sperm samples (2 × 10^6^/mL) were stained with 1 × JC-1(10 μg/mL) probe at 37°C for 30 min in the dark. The sperm samples were centrifuged at 600 × *g* for 5 min, washed, and resuspended with JC-1 working solution. The stained sample was placed on ice before analysis (within 5 min). Fluorescence intensity of both mitochondrial JC-1 monomers (λex 514 nm, λem 529 nm) and aggregates (λex 585 nm, λem 590 nm) were detected using a monochromator microplate reader (Safire II, Tecan, Switzerland). The Δψm of sperm in each treatment group was calculated as the fluorescence ratio of red (aggregates) to green (monomers). The samples were also monitored and photographed under an epifluorescence microscope. Sperm with red fluorescence indicated sperm with high mitochondrial membrane potential (HMMP), while sperm with low mitochondrial membrane potential (LMMP) were green (Supplementary Figure 1C). Analyses were performed in triplicate (*n* = 3).

### Assessment of Sperm LDH, MDH and SDH Activities

The LDH, MDH, SDH activities were measured using Lactate Dehydrogenase assay kit, Malate Dehydrogenase assay kit, Succinate Dehydrogenase assay kit (Nanjing Jiancheng Bioengineering Institute, China), respectively. According to the manufacturer’s instructions, sperm sample pellets were suspended with PBS at a concentration of 1.0 × 10^8^ sperm/mL, then lysed ultrasonically (20 kHz, 750 W, operating at 40% power, 5 cycles of 3 s on and 5 s off) and centrifuged at 2000 × *g* for 10 min at 4°C. The supernatants were added to a 96-well plate for the analysis of LDH, MDH and SDH activities using a microplate reader at 450, 340, and 600 nm respectively. The LDH, MDH and SDH activities were expressed as mU per mg protein. Protein concentrations were determined using Bradford’s method with BSA as the standard. Analyses were performed in triplicate (*n* = 3).

### Measurement of ATP Content

Sperm ATP content was measured using an ATP Assay Kit (Beyotime Institute of Biotechnology). According to our previous study (Zhu et al., 2018), samples were lysed with lysis buffer followed by sonication (20 kHz, 750 W, operating at 40% power, 5 cycles of 3 s on and 5 s off) and centrifuged at 12 000 × *g* for 10 min. The supernatant was used to analyze the ATP level, 50 μL of the sample was added to the 100 μL luciferin/luciferase reagent in 96-well plates. The luminescence at integration × 1000 ms was read using an Ascent Luminoskan luminometer (Thermo Scientific, Palm Beach, FL, United States) with BPSE as a blank for each experiment. Standards were prepared from ATP standard using serial dilutions to obtain concentrations of 0.01, 0.03, 0.1, 0.3, 1, 3, 10 μM. Analyses were performed in triplicate (*n* = 3).

### Immunofluorescence

Sperm were isolated from goat epididymis of caput, corpus and cauda. Samples were fixed with 4% paraformaldehyde for 10 min at room temperature after washed for three times in PBS. The samples were permeabilized with 0.5% Triton X-100 in PBS for 10 min after washed for three times with PBS (5 min each time). Non-specific binding was blocked with PBS supplementation of 10% BSA (Sigma-Aldrich) for 30 min at room temperature. Samples were then incubated overnight at 4°C with anti-GSK3α/β (1:100, sc-7291, Santa Cruz Biotechnology, Santa Cruz, CA, United States). The negative control was treated without anti-GSK3α/β. Next day, the sperm were washed three times in PBS and incubated with goat anti-mouse (1:100, sc-516141, Santa Cruz Biotechnology) antibody for immunofluorescence labeling. After labeling, sperm samples were washed twice with PBS and then analyzed with a flow cytometer (FAC SCalibur, BD Biosciences) with excitation at 525 nm and emission at 590 nm (BL2). And we also used DAPI (CWBIO) and FITC-PNA to counterstained with the sample after FACS analyze. Fluorescent images were captured with fluorescence microscopy (80i, Nikon).

### Western Blotting

Samples were lysed in RIPA solution (R0010, Solarbio), centrifuged at 12,000 × *g* for 30 min at 4°C and mixed with SDS lysates loading buffer and boiled for 5 min at 100°C. Cell lysates were separated by SDS-PAGE and transferred to PVDF membranes (Millipore). The membranes were incubated with the following primary antibodies: anti-GSK3α/β (sc-7291), anti-phospho-GSK3α/β (Ser21/9) (#9327; Cell Signaling Technology, lnc., Danvers, MA, United States), anti-α-tubulin (sc-398103, Santa Cruz Biotechnology), anti-β-actin (#3700; Cell Signaling Technology, lnc., Danvers, MA, United States) and anti-CD9 (#13403; Cell Signaling Technology, lnc., Danvers, MA, United States) (1:1000). Secondary antibodies were horseradish peroxidase-linked anti-mouse antibody (1:5000; ab205719; Abcam) and horseradish peroxidase-linked anti-rabbit antibody (#7074; Cell Signaling Technology, lnc., Danvers, MA, United States). The membranes were visualized on a Bio-Rad Chemidoc XRS using a Western Bright ECL Kit (WBKLS0500; Merck, Germany).

### Sperm-Zona Pellucida-Binding Assay

Mature ovulated oocytes were collected from the oviduct of immature mice (3-weeks old) after injecting with 4 IU of eCG for 48 h followed by 5 IU of hCG for 16 h. According to the previous studies (Bromfield et al., 2014; Zhu et al., 2018), ten oocytes were placed in a 50 μL droplet of fertilization medium for each group. Then 50 μL of the incubated sperm suspension was added into a fertilization medium droplet containing 10 oocytes and incubated for 3 h at 39°C in a humidified atmosphere saturated with 5% CO_2_. Following incubation, the total number of sperms tightly bound to each of zona pellucida was counted.

### Experiment Design

Experiment 1 was designed to identify the localization of GSK3α/β in goat sperm, and to analyze the expression of GSK3α/β in goat sperm during the process of sperm maturation in epididymis via immunofluorescence.

Experiment 2 was performed to evaluate whether GSK3α/β regulates goat sperm motility via phospho-Ser21-GSK3α and phospho-Ser9-GSK3β using the motility activator and GSK3α/β inhibitor. In our previous study (Zhu et al., 2018), it was observed that AICAR could activate goat sperm motility patterns. Thus, we used AICAR as an activator of goat sperm motility in the present study. Different doses of AICAR (0, 1, 2, and 4 mM) and CHIR99021 (0, 6.7, 67, and 670 nM) were incubated with goat sperm. It was observed that the progressive motility was only improved in the 2 mM AICAR and 67 nM CHIR99021 treatments compared to the control, meanwhile both 4 mM AICAR and 670 nM CHIR99021 decreased it (Supplementary Figure 2A). Moreover, the sperm viability was no significantly changed among the treatments of 0, 1, and 2 mM AICAR or 0, 6.7, 67 nM CHIR99021, but decreased in the 4 mM AICAR and 670 nM CHIR99021 (Supplementary Figure 2B), which suggested that the 4 mM AICAR and 670 nM CHIR99021 were toxic to sperm. Therefore, we treated sperm with 2 mM AICAR and 67 nM CHIR99021 in the following experiments. Specifically, three groups were evaluated: (1) a group incubated with 2 mM AICAR, an activator of motility; (2) a group incubated with 67 nM CHIR99021, a specific inhibitor of GSK3α/β; and (3) a control group, incubated without CHIR99021 and AICAR. Sperm motility, membrane integrity and serine phosphorylation of GSK3α/β were analyzed.

As GSK3α/β was strongly localized with the peri-acrosomal region of mature sperm in Experiment 1, Experiment 3 was designed to evaluate whether the GSK3α/β kinase plays a role in regulating the sperm acrosome reaction. Acrosome reaction was induced *in vitro* with capacitation medium in the presence of 10 μM calcium ionophore A23817. Three treatments were conducted in experiment 3: (1) sperm with 10 μM A23817; (2) sperm with 67 nM CHIR99021; and (3) sperm without A23817 or CHIR99021 in non-capacitation conditions (control). Sperm acrosome reaction, motility, mitochondrial membrane potential, serine phosphorylation of GSK3α/β and sperm-zona pellucida binding capacity were analyzed.

Experiment 4 was to examine whether GSK3α/β regulates sperm motility and acrosome reaction via energy metabolism. ATP content, activities of LDH, MDH and SDH were analyzed in Experiments 2 and 3.

### Statistical Analysis

All data were tested for normality and variance homogeneity prior to statistical analysis. Data were transformed by arc-sin square root transformation when it is necessary. All data were analyzed by one-way ANOVA, and multiple comparisons with Tukey test was performed using SPSS version 17.0 for Windows (SPSS Inc., Chicago, IL, United States). All the values are presented as mean ± standard error of the mean (SEM). Treatments were considered statistically different from one another at *p* < 0.05.

## Results

### Expression and Location of GSK3α/β in Mature Goat Sperm

**FIGURE 1:**
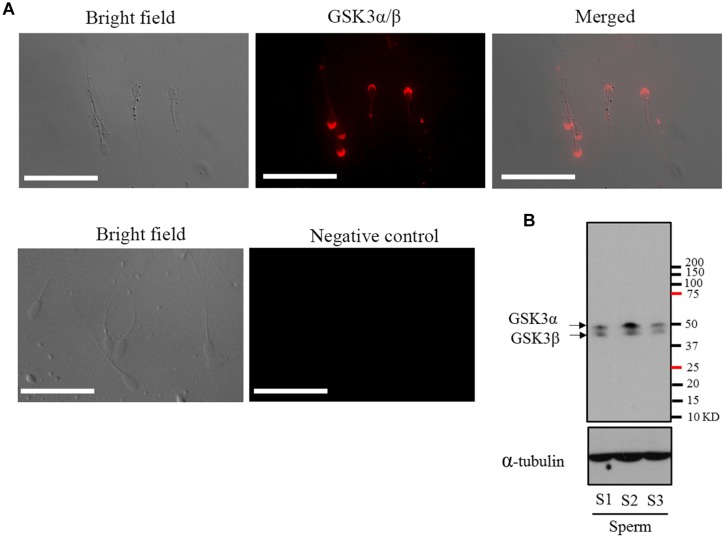
**(A)** Detection and immunofluorescent localization of GSK3α/β in goat sperm. Sperm with red fluorescence indicated GSK3α/β was present in the peri-acrosomal domain, in the mid-piece, and principal piece of the tail. **(B)** Western blotting analysis of GSK3α/β in goat mature sperm. Bars = 30 μm. S1, sperm sample 1; S2, sperm sample 2; S3, sperm sample 3.

The presence of GSK3α/β in mature goat sperm was investigated by immunofluorescence and Western blotting methods using specific antibody. As shown in Figure 1A, GSK3α/β was highly present in the peri-acrosomal domain, as well as in the mid-piece and principal piece of the tail. It was observed that two predominant bands of 51 and 47 kDa representing both the α and β (respectively) isoforms of GSK3 protein were present in mature goat sperm (Figure 1B).

### Sperm GSK3α/β Was Increased During the Process of Sperm Maturation in the Epididymis

**FIGURE 2:**
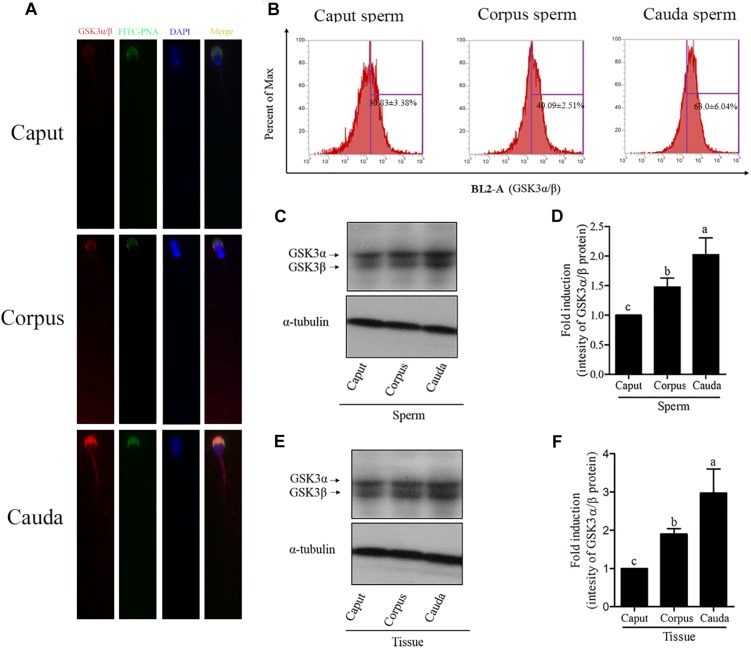
Detection of the GSK3α/β expression in goat epididymal sperm by immunofluorescence **(A,B)** and western blotting **(C,D)**. **(E,F)** Detection of the GSK3α/β expression in epididymal tissue. Values are specified as mean ± standard error of the mean (SEM). Columns with different lowercase letters differ significantly (*p* < 0.05).

The GSK3α/β protein was present in functionally immature caput and proximal corpus, cauda epididymal sperm, which presented with an increase from caput to cauda (Figures 2A,B). The western blotting results were also in accordance with those results (Figures 2C,D). Interestingly, when we counterstained with FITC-PNA, a marker of the sperm acrosome, it was observed that the GSK3α/β was also colocalized with FITC-PNA in the peri-acrosomal region of all sperm types (Figure 2A), which indicated that GSK3α/β might be related with sperm acrosome reaction.

Since haploid sperm are incapable of *de novo* gene transcription and protein translation, and the increase of GSK3α/β was unexpected during the process of sperm maturation, the additional GSK3α/β might be acquired by sperm during the epididymal transport. We detected the GSK3α/β level in different tissues of the goat epididymis (caput, corpus and cauda). As shown in Figures 2E,F, the level of GSK3α/β was also increased from the caput to cauda epididymis. Moreover, it was observed that the GSK3α/β was expressed in the epididymosomes (Supplementary Figure 3).

### GSK3α/β Regulates Sperm Motility via Phospho-Ser21-GSK3α and Phospho-Ser9-GSK3β

**FIGURE 3:**
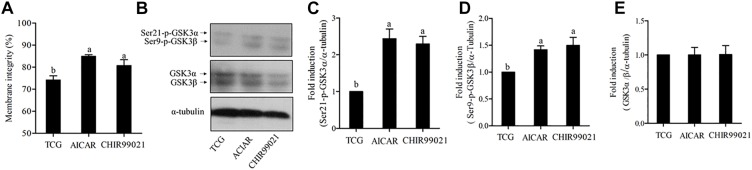
Sperm membrane integrity **(A)** was detected after 3 h of incubation. The levels of phospho-ser21-GSK3α, phospho-ser9-GSK3β, and GSK3α/β were analyzed by western blotting after 3 h of incubation **(B)**. The blots were normalized to an endogenous protein (α-tubulin) **(C–E)**. Values are specified as mean ± standard error of the mean (SEM). Columns with different lowercase letters differ significantly (*p* < 0.05).

To test whether GSKα/β regulates sperm motility, a cell-permeable highly selective GSK3α/β inhibitor (CHIR99021) and sperm motility activator (AICAR) were used in this study. Caudal epididymis sperm were incubated with TCG (control); TCG and 2 mM AICAR; TCG and 67 nM CHIR99021 for 3 h. Compared to the control, sperm incubated with AICAR and CHIR99021 significantly increased the percentage of progressive motility (Table 1). In addition, as shown in Table 1, an increase in the VCL, VSL, VAP, BCF, LIN were observed in the treatments with supplementation of AICAR and CHIR99021. We also detected sperm membrane integrity, which is essential for motility. Supplementation of AICAR and CHIR99021 significantly increased the value of membrane integrity (Figure 3A).

**TABLE 1:** Effects of AICAR and CHIR99021 on goat sperm motility parameters measured with CASA during incubation for 3 h.

**Sperm parameters**	**TCG**	**AICAR**	**CHIR99021**
Total motility (%)	86.7 ± 0.9^a^	86.8 ± 1.0^a^	84.8 ± 0.7^a^
Progressive motility (%)	51.4 ± 1.6^b^	64.8 ± 1.2^a^	62.0 ± 0.7^a^
VCL (μm/s)	92.4 ± 3.5^b^	122.7 ± 2.3^a^	113.4 ± 5.9^a^
VSL (μm/s)	28.1 ± 1.1^b^	58.6 ± 1.1^a^	62.2 ± 5.6^a^
VAP (μm/s)	31.2 ± 1.5^b^	35.3 ± 1.7^a^	34.6 ± 0.4^a^
BCF (Hz)	22.3 ± 0.4^b^	24.3 ± 0.6^a^	23.7 ± 0.5^*ab*^
LIN (%)	26.6 ± 0.8^b^	49.5 ± 1.3^a^	52.9 ± 3.5^a^
WOB (%)	30.3 ± 0.3^a^	25.4 ± 1.0^b^	26.5 ± 3.3^b^

*Values are expressed as mean ± standard error of the mean (SEM). Different letters within column indicate significant difference (*p* < 0.05). VCL, curvilinear velocity; VSL, straight-line velocity; VAP, average path velocity; BCF, beat-cross frequency; LIN, linearity; WOB, wobble.*

To test whether both isoforms of GSK3α/β were participated in regulating sperm motility, we evaluated the phosphorylation of ser21-GSK3α and of ser9-GSK3β under the incubation conditions. The results showed a significant increase in the phosphorylation of the isoform α at ser21 incubation with AICAR and CHIR99021 (Figures 3B,C). Interestingly, the results of phosphorylation of GSK3β at ser9 were similar to those of ser21 of GSK3α (Figures 3B,D). However, the total level of GSK3α/β was not significantly changed among the treatments during 3 h of incubation (Figures 3B,E).

### GSK3α/β Regulates Sperm Acrosome Reaction via Phospho-Ser21-GSK3α and Phospho-Ser9-GSK3β

**FIGURE 4:**
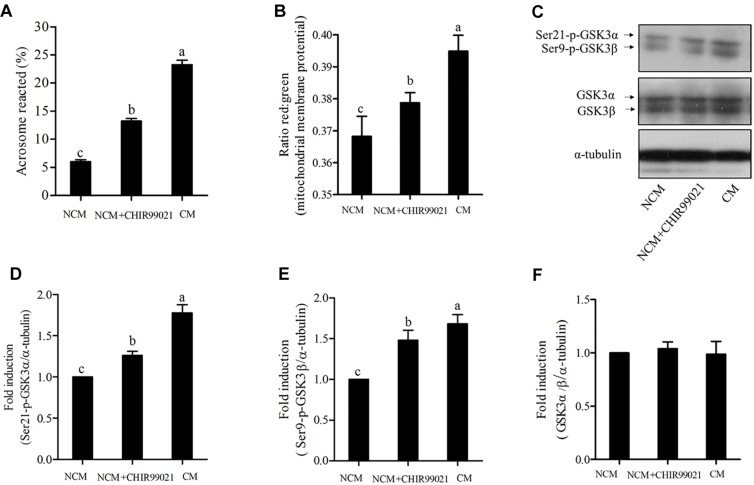
Sperm acrosome reaction **(A)** and mitochondrial membrane potentials **(B)** were detected after acrosome reaction-induced. The levels of phospho- ser21-GSK3α, phospho-ser9-GSK3β, and GSK3α/β were analyzed by western blotting after acrosome reaction-induced **(C)**. The blots were normalized to an endogenous protein (α-tubulin) **(D–F)**. Values are specified as mean ± standard error of the mean (SEM). Columns with different lowercase letters differ significantly (*p* < 0.05). NCM, non-capacitation medium; CM, capacitation medium.

As GSK3α/β was colocalized with FITC-PNA in the peri-acrosomal region of all sperm types (Figure 1A), we used GSK3α/β inhibitor, CHIR99021, to detect whether GSK3α/β regulates sperm acrosome reaction. As shown in Figure 4A, the addition of CHIR99021 significantly increased the percentage of sperm that had undergone acrosome reaction (*p* < 0.05). Sperm parameters were also increased by the addition of CHIR99021 to the NCM (Table 2). Compared to the NCM treatment, progressive motility, VAP, BCF, VCL, VSL, VAP and WOB were much higher in the treatments with capacitation medium or CHIR99021. Moreover, mitochondrial membrane potential was also measured as it was correlated with acrosome reaction. The value of mitochondrial membrane potential was increased by the addition of CHIR99021 in Figure 4B. When we evaluated the phospho-GSK3α/β (Ser21/9) of sperm, it was observed that both the phospho-ser21-GSK3α and phospho-ser9-GSK3β were also significantly increased in capacitation medium and CHIR99021 treatments (Figures 4C–E). Meanwhile, the total level of GSK3α/β was not significantly altered by those treatments (Figures 4C,F).

**TABLE 2:** Effects of GSK3α/β inhibitor on goat sperm motility parameters measured with CASA during acrosome reaction.

**Sperm parameters**	**NCM**	**CM**	**NCM+CHIR99021**
Total motility (%)	83.8 ± 1.7^b^	87.1 ± 0.5^a^	87.8 ± 0.4^a^
Progressive motility (%)	46.3 ± 1.9^c^	72.2 ± 0.5^a^	57.8 ± 0.8^b^
VCL (μm/s)	79.3 ± 1.4^c^	135.2 ± 1.6^a^	102.5 ± 1.5^b^
VSL (μm/s)	26.5 ± 0.8^c^	49.8 ± 0.7^a^	33.4 ± 1.2^b^
VAP (μm/s)	26.2 ± 0.6^c^	47.4 ± 0.9^a^	35.7 ± 0.6^b^
BCF (Hz)	21.5 ± 0.3^c^	27.3 ± 0.3^a^	24.8 ± 0.3^b^
LIN (%)	26.5 ± 0.6^c^	39.1 ± 0.7^a^	29.9 ± 0.8^b^
WOB (%)	28.4 ± 0.4^b^	32.5 ± 0.4^a^	31.9 ± 0.3^a^

*Values are expressed as mean ± standard error of the mean (SEM). Different letters within column indicate significant difference (*p* < 0.05). VCL, curvilinear velocity; VSL, straight-line velocity; VAP, average path velocity; BCF, beat-cross frequency; LIN, linearity; WOB, wobble.*

Moreover, when we evaluated the effect of CHIR99021 on sperm-zona pellucida binding capacity (a potential indicator for sperm fertilization), it was observed that addition of either CHIR99021 or AICAR significantly increased the number of zona-pellucida-bound sperm, compared to the control (Supplementary Figure 4).

### GSK3α/β Affects Energy Metabolism

**FIGURE 5:**
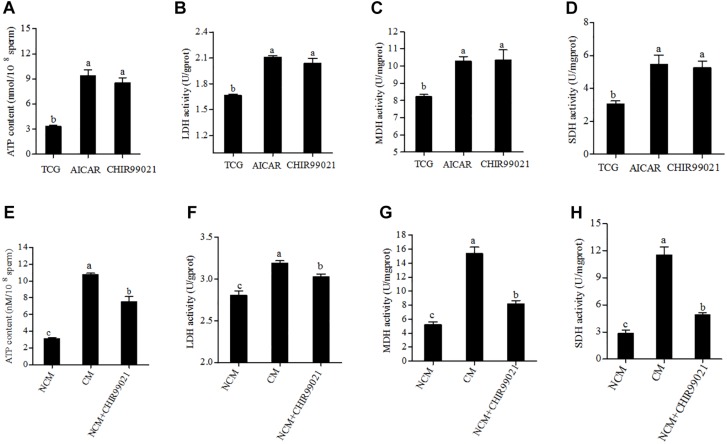
Effects of AICAR and CHIR99021 on goat sperm ATP content, LDH, MDH, and SDH activities during 3 h of incubation at 37°C **(A–D)**. Effects of CHIR99021 on ATP content, activities of LDH, MDH, and SDH during sperm induced to acrosome reaction **(E–H)**. Values are specified as mean ± standard error of the mean (SEM). Columns with different lowercase letters differ significantly (*p* < 0.05). NCM, non-capacitation medium; CM, capacitation medium.

Glycolysis and oxidative phosphorylation are two pathways for ATP synthesis that essential to sperm motility and acrosome reaction. To investigate the mechanism of GSK3α/β regulates sperm motility and acrosome reaction, we measured the ATP content. As shown in Figure 5A, the level of ATP content in the treatment with AICAR or CHIR99021 was much higher than the control. During the capacitation process, though the value of ATP content in capacitation treatment showed the highest, the treatment added with CHIR99021 also presented a higher level of ATP than the NCM treatment (Figure 5E). It is well known that the LDH is essential for glycolysis, while MDH and SDH are the rate-limiting enzymes of oxidative phosphorylation. During 3 h of incubation, the addition of either CHIR99021 or AICAR significantly increased the activities of LDH, MDH, and SDH (Figures 5B–D). As shown in Figures 5E–H, the results of activities of LDH, MDH, and SDH were similar to that of ATP content after sperm induced with acrosome reaction. Sperm induced acrosome reaction showed much higher value in the activities of LDH, MDH, and SDH. Additionally, it was observed that sperm treated with CHIR99021 significantly increased those rate-limiting enzymes activities, compared with NCM treatment (Figures 5E–H).

## Discussion

The present study demonstrated for the first time that GSK3α/β was expressed in goat sperm, especially in the peri-acrosomal, mid-piece and principal piece of the tail. Using the AICAR to activate sperm motility patterns or addition of CHIR99021 significantly increased the phospho-ser21-GSK3α and phospho-ser9-GSK3β. Moreover, it was observed that the addition of CHIR99021 significantly increased the percentage of sperm with acrosome reaction. The value of ATP content, activities of the metabolism enzymes (LDH, MDH, and SDH) were also increased with the treatment of CHIR99021. These data indicated that the GSK3α/β protein regulated sperm motility and acrosome reaction through affecting energy metabolism in goat sperm.

GSK3α/β has been identified in the sperm of mammalian species including bull (Vijayaraghavan et al., 1997, 2000; Somanath et al., 2004), boar (Aparicio et al., 2007), and mouse (Bhattacharjee et al., 2015; Koch et al., 2015; Reid et al., 2015; Zhu et al., 2016). Previous studies reported that the GSK3α/β was present in the tail of bovine sperm (Vijayaraghavan et al., 2000) whereas it was presented in the sperm head and mitochondria in mouse sperm (Reid et al., 2015; Zhu et al., 2016). However, in the present study, the GSK3α/β protein was highly distributed in the peri-acrosomal, mid-piece, and principal piece of the tail in goat sperm. The difference in the GSK3α/β distributions in sperm might be due to the species-specific differences.

Mammalian sperm are generated in the seminiferous tubules of the testis and become mature in epididymis. Within the epididymis, sperm are embedded in the intraluminal fluid that contains different proteins, which interact with the sperm membrane surface (Dacheux et al., 2005). The epididymal epithelia undergo apocrine secretion at its apical pole (Hermo and Jacks, 2002). This secretion involves the formation of epididymosomes, which are rich in cholesterol, proteins and nuclear acids (Sullivan and Saez, 2013). During sperm transit along the epididymis, a lot of epididymal-secreted proteins are transferred to the sperm mediated by epididymosomes (Sullivan et al., 2005). Interestingly, in the present study, the western immunoblotting and immunofluorescence analysis data showed that the relative amount of sperm GSK3α/β was increased as sperm moving through the epididymis. Moreover, it was also observed that the GSK3α/β protein was expressed in the goat epididymosomes (Supplementary Figure 3) in the present study. Reid et al. (2015) reported that mouse sperm acquired the additional GSK3 within the male reproductive tract via direct interaction of sperm heads with extracellular structures known as epididymal dense bodies. In the present study, we showed that the total level of sperm GSK3α/β increased during maturation in the epididymis. In addition, the GSK3α/β protein is also distributed in the goat epididymosomes. Therefore, we speculated that goat sperm, as mouse sperm, probably acquire GSK3α/β from the epididymosomes during maturation in the epididymis. Future study is needed to prove it.

During transit along epididymis, a series of morphological, biochemical and physiological changes occur and finally sperm acquire progressive motility (Cornwall, 2009). In the previous study (Somanath et al., 2004), the serine phosphorylation of GSK3α/β was increased during the bovine sperm passage through the epididymis, and it was observed that pharmacological stimulation of motility *in vitro* also caused an increase in GSK3α/β serine phosphorylation. Similarly, Aparicio et al. (2007) found that the boar sperm motility was regulated by serine phosphorylation of GSK3α. Moreover, when Bhattacharjee et al. (2015) examined the functions of GSK3α in male fertility using a *Gsk3α* knockout mouse model, it was observed that the female is unaffected, but the sperm motility parameters in male were impaired in *Gsk3α* mutant mouse. In the present study, the treatments with either motility effector (AICAR) or GSK3α/β inhibitor (CHIR99021) could cause an increase in goat sperm motility patterns, phospho-ser21-GSK3α as well as phospho-ser9-GSK3β. Collectively, GSK3α/β serine phosphorylation is suggested to regulate sperm motility. A comprehensive analysis of the proteomic composition of mouse acrosome has revealed that tremendous changes occurred when compared caput with caudal sperm (Guyonnet et al., 2012). It was observed that the GSK3α/β protein was strongly localized in goat sperm acrosome, and interestingly, the abundance of GSK3α/β in the sperm acrosome was increased during the transit through the epididymis in this study. Such elevated GSK3α/β may indicate its potential role for acrosome reaction and fertility. Indeed, we found that the addition of CHIR99021 led to an increase in sperm acrosome reaction and total number of sperms bound to the zona pellucida. Furthermore, it has been reported that the GSK3β protein mediated acrosome reaction in bovine sperm (Belenky and Breitbart, 2017). However, Aparicio et al. (2007) reported that inhibition of GSK3 activity did not cause any changes in the number of acrosome-reacted porcine sperm. The contradictory findings may be due to species-specific differences.

It is well known that ATP is essential for sperm motility and acrosome reaction (Stival et al., 2016). Bhattacharjee et al. (2015) demonstrated that the sperm ATP level was lower in *Gsk3α* mutant mice when compared with wild type mice. In this study, the addition of GSK3 inhibitor, CHIR99021, led to an increase of ATP content in goat sperm, suggesting that the GSK3α/β is involved in regulating the ATP production in goat sperm. As both glycolysis and oxidative phosphorylation pathways could generate ATP in sperm (du Plessis et al., 2015), activities of the enzymes involved in those pathways are essential for the ATP generation. When we analyzed the activities of LDH (an important enzyme for glycolysis), MDH and SDH (two rate-limiting enzymes for oxidative phosphorylation), it was observed that addition of CHIR99021 significantly increased the activities of LDH, MDH, SDH, and that induced-acrosome reaction *in vitro*. Most of the enzymes involved in glycolysis pathway are located in the sperm tail and acrosome (du Plessis et al., 2015); while the MDH and SDH enzymes involved in the tricarboxylic acid (TCA) cycle that located in sperm mitochondria (Piomboni et al., 2012). It has been reported that the GSK3α/β protein existed not only in the cytosol, but also in the mitochondria of somatic cells (Yost et al., 1998; Bijur and Jope, 2003) and mouse sperm mitochondria (Zhu et al., 2016). Moreover, in present study, the GSK3α/β protein was observed at the mid-piece in which the mitochondria are located (Amaral et al., 2013), suggesting that the GSK3α/β protein maybe existed in the mitochondria of goat sperm. Importantly, in the present study when sperm were treated with GSK3α/β inhibitor, the activities of LDH, MDH and SDH were significantly enhanced, indicating mitochondrial energy metabolism was changed. As both the GSK3α/β protein and the TCA cycle enzymes (MDH and SDH) locate in the sperm mid-piece, the GSK3α/β protein may regulate in directly or indirectly those enzyme activities. It would be interesting to uncover the mechanism of how GSK3α/β regulates the mitochondrial energy metabolism in sperm.

## Conclusion

**FIGURE 6:**
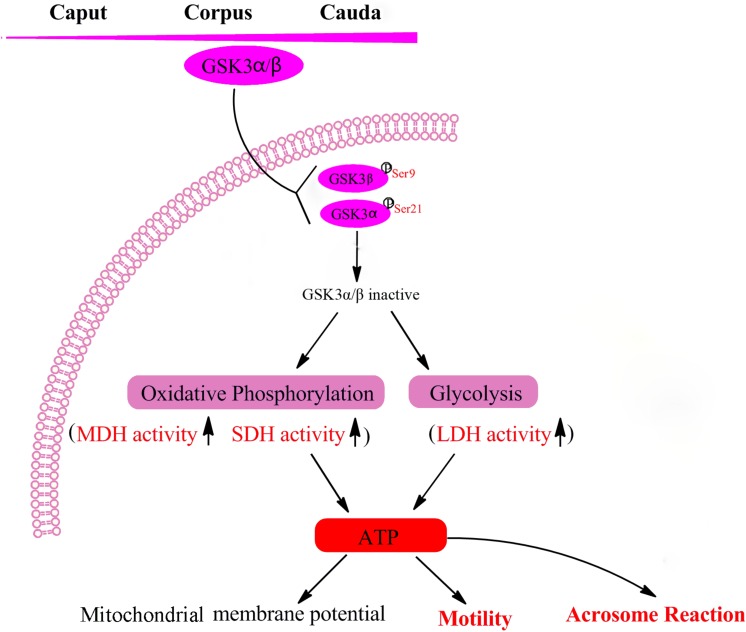
Mechanisms of GSK3α/β regulate sperm motility and acrosome reaction. Sperm GSK3α/β is accumulated during the maturation in the epididymis. The phosphorylation of GSK3α/β led to GSK3α/β inactive, which resulted in enhancing sperm energy metabolism involved in oxidative phosphorylation and glycolysis, thus regulating sperm function. MDH, malate dehydrogenase; SDH, succinate dehydrogenase; LDH, lactate dehydrogenase; ATP, adenosine triphosphate.

In conclusion, the level of GSK3α/β in goat sperm was increased during transit from caput to caudal epididymis. GSK3α/β serine phosphorylation regulated goat sperm motility and acrosome reaction via mediating of energy pathways in glycolysis and oxidative phosphorylation (Figure 6). Our future work will focus on the application of GSK3α/β in sperm preservation to promote fertility.

## Data Availability

The raw data supporting the conclusions of this manuscript will be made available by the authors, without undue reservation, to any qualified researcher.

## Ethics Statement

All animal and experimental procedures were approved by the Northwest A&F University Institutional Animal Care and Use Committee.

## Author Contributions

ZZ designed the study, contributed to all experiments, and wrote the manuscript. RL contributed to the analysis of acrosome reaction. LW and YZ participated in the interpretation of the data. SH edited the manuscript. YL was responsible for the discussion about the experimental design and data analysis, and edited the manuscript. WZ conceived and designed the study.

## Conflict of Interest Statement

The authors declare that the research was conducted in the absence of any commercial or financial relationships that could be construed as a potential conflict of interest.
