# Supplementary material for: Glycogen Synthase Kinase-3 Regulates Sperm Motility and Acrosome Reaction via Affecting Energy Metabolism in Goats
Source: Front Physiol. 2019 Jul 30;10:968. doi: 10.3389/fphys.2019.00968 (PMC6682598; doi:10.3389/fphys.2019.00968)
Supplement: Supplementary file 1 [file Table_1.DOCX]

**Supplementary Information**


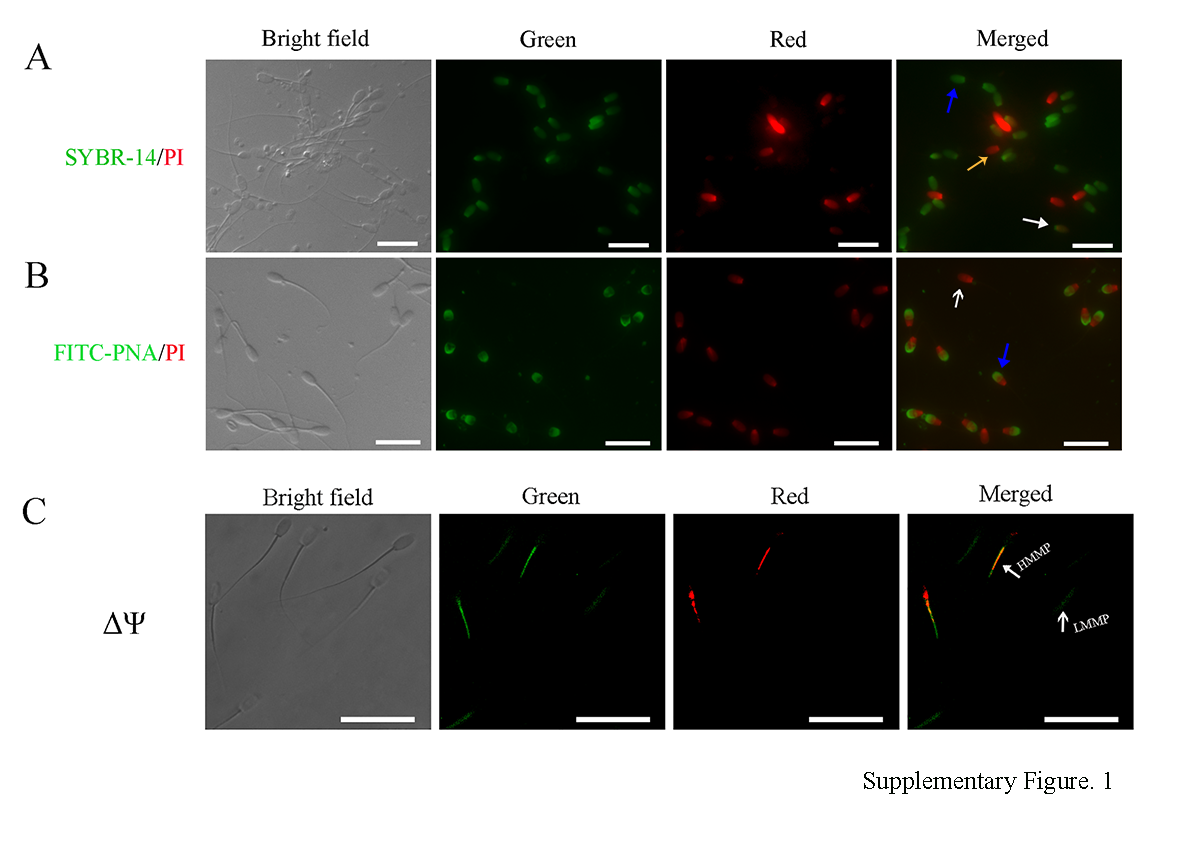


Supplementary Figure 1. Sperm membrane integrity (A), acrosome reaction (B) and mitochondrial membrane potential (C) were detected by SYBR-14/PI, FITC-PNA/PI and JC-1 staining, respectively. A: blue arrow indicated sperm with membrane integrity, yellow arrow indicated sperm with membrane damage, white arrow indicated sperm with membrane slightly damage. B: blue arrow indicated sperm with acrosome no-reaction, white arrow indicates sperm with acrosome reaction. HMMP: high mitochondrial membrane potential, LMMP: low mitochondrial membrane potential. Bars=30 μm.


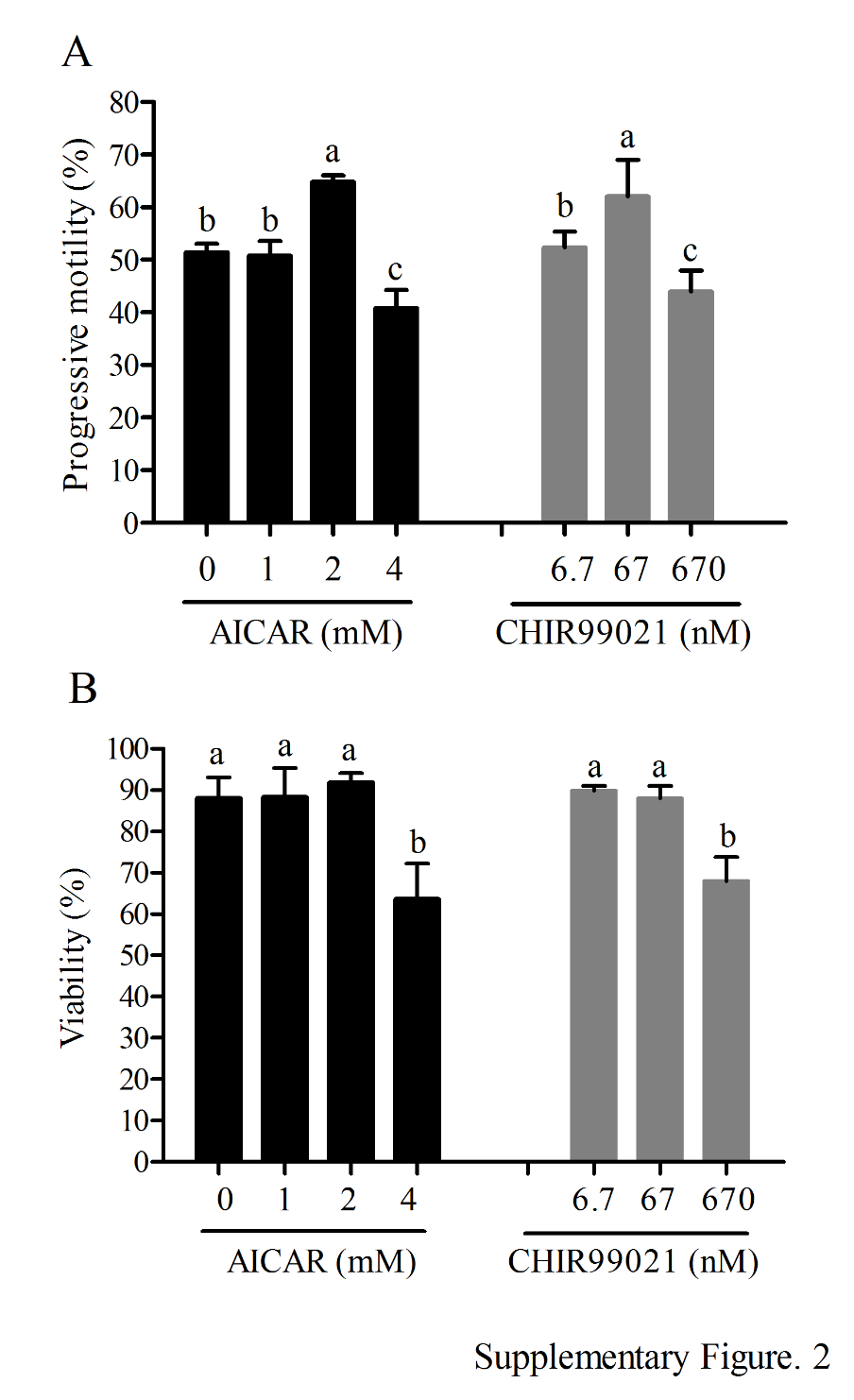


Supplementary Figure 2. Effect of different doses of AICAR (0, 1, 2, 4 mM) and CHIR99021 (0, 6.7, 67, 670 nM) on goat sperm progressive motility(A) and viability (B). Values are specified as mean ± standard error of the mean (SEM). Columns with different lowercase letters differ significantly (p<0.05).


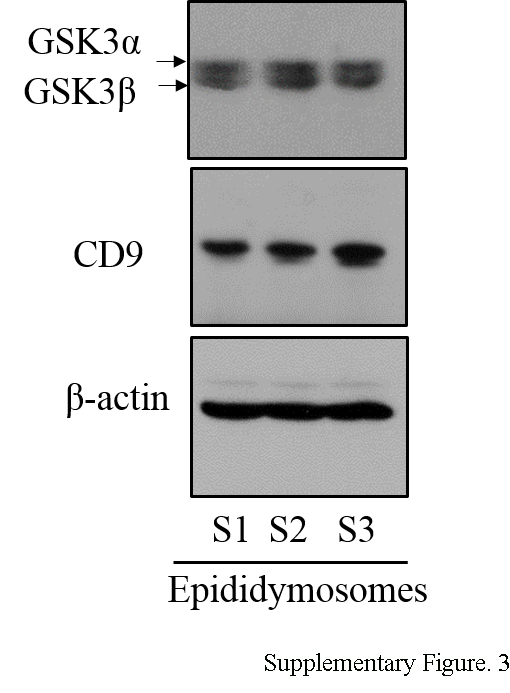


Supplementary Figure 3. Identification of the expression of GSK3α/β in epididymosomes by western blotting. CD9, a marker of epididymosomes. S1, sample 1; S2, sample 2; S3, sample 3.


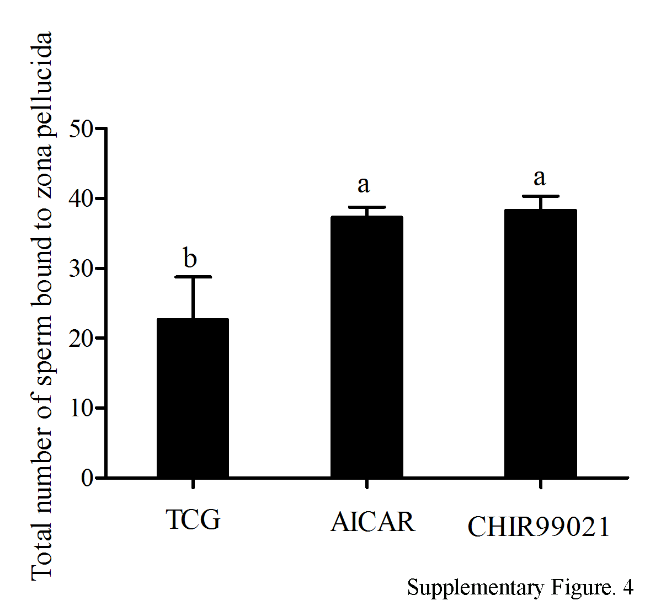


Supplementary Figure 4. Effect of CHIR99021 and AICAR on sperm-zona pellucida binding capacity. Values are specified as mean ± standard error of the mean (SEM). Columns with different lowercase letters differ significantly (p<0.05).
